# Impact of COVID-19 acute respiratory disease on the risk factors attributed to cancer patients

**DOI:** 10.1016/j.toxrep.2021.12.004

**Published:** 2021-12-16

**Authors:** Elena Lak, Mohammad Javad Mohammadi, Homayon Yousefi

**Affiliations:** a Assistant Professor of Gastroenterology and Hepatology, Department of Internal Medicine, School of Medicine, Ahvaz Jundishapur University of Medical Sciences, Ahvaz, Iran; b Department of Environmental Health Engineering, School of Health AND Air Pollution and Respiratory Diseases Research Center, Ahvaz Jundishapur University of Medical Sciences, Ahvaz, Iran; c Assistant Professor of Pediatric Hematology and Oncology, Department of Internal Medicine, School of Medicine AND Thalassemia & Hemoglobinopathy Research center, Health research Institute, Ahvaz Jundishapur University of Medical Sciences, Ahvaz, Iran

**Keywords:** COVID-19, coronavirus disease 2019, SARS-CoV-2, severe acute respiratory syndrome coronavirus 2, COVID-19 ARD, acute respiratory disease, CDs, Communicable diseases, WHO, World Health Organization, PCR, Real-time Polymerase Chain Reaction, LUAD, Lung adenocarcinoma, KICH, Kidney Chromophobe, PRAD, Prostate adenocarcinoma, ESCA, Esophageal carcinoma, UCEC, Uterine Corpus Endometrial Carcinoma, KIRP, Kidney renal papillary cell carcinoma, CCC-19, COVID-19 and Cancer Consortium, AACR, American Association of Cancer Research, ASCO, American Society of Clinical Oncology, ESMO, European Society for Medical Oncology, ICU, Intensive Care Unit, COVID-19, Communicable diseases, Cancer, Acute respiratory disease, Risk factors, Health

## Abstract

Communicable diseases (CDs) based on Health organization reported are one of the most threat for human health. SARS-CoV-2 (severe acute respiratory syndrome coronavirus 2) is the main pandemic that nowadays it threatens the health of people around the world, especially cancer patients. The purpose of this study was to investigate the effects of COVID-19 acute respiratory disease (COVID-19 ARD) on risk factors related to health of cancer patients. A review study of was conducted to base on results of various studies published. Nine hundred and eighty articles were retrieved based on various databases: Science Direct, Taylor & Francis, Google Scholar, Elsevier, PubMed and BMJ. In this study, were used the results of research on COVID-19 and its effects on risk factors attributed to cancer patients. The literature signs a notable undesirable affect from COVID-19 on risk factors attributed to health of cancer patients.

Result showed that transfer SARS-CoV-2 viruses can endanger health of cancer patients due to interruption of the disease treatment process and increase number of deaths between in this patents.

The survey requires the need to act creating healthy conditions to continue the treatment process and vaccination coverage among these patients in order to decrease the transmission of COVID-19 acute respiratory disease and increase the success rate of cancer treatment.

## Highlights

• Clinical features of COVID-19 acute respiratory disease (ARD).
• SARS−COV-2 has created many problems in providing health care to cancer patients.
• SARS−COV-2 causes more severe complications in cancer patients.

## Graphical abstract

**Figure:**
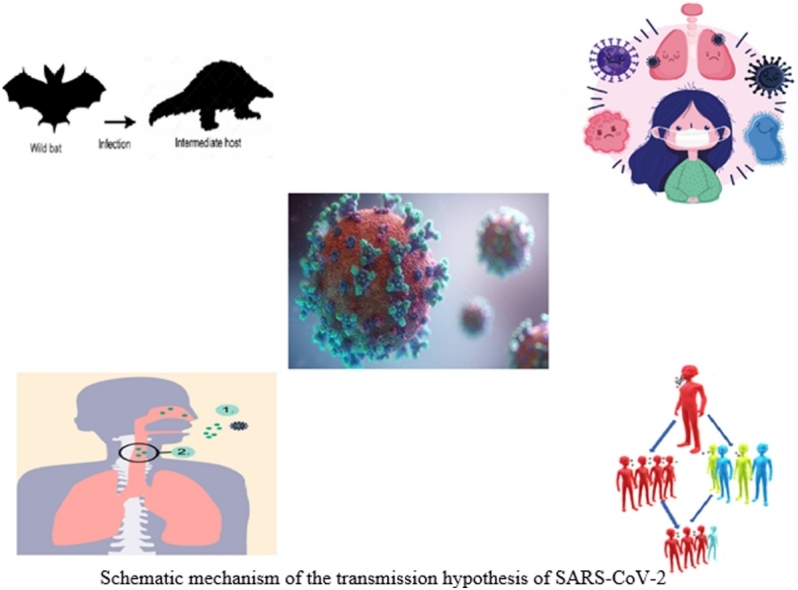

## 1 Introduction

In 2019, a new unprecedented coronavirus called COVID-19 acute respiratory disease emerged in Wuhan, Chain, rapidly spreads from country to country, and became a global crisis, impacting every aspect of human life, economic devastation and social anxiety around the world [[1], [2], [3]]. In the world more than 180 countries have affected COVID-19 outbreak [4,5].

In more than 212 countries and territories around the world confirmed COVID-19 cases. According to reported the World Health Organization (WHO) declared SARS−COV-2 is has become one of the most prevalent diseases in the world [6].

Despite the rapid global spread of the COVID-19, complications and clinical evidence of this disease are still unclear [7,8]. The reported rate of transmission of symptomatic infections varies with location and infection control interventions [9].

Based on reports and results of various studies in different parts of the world, the most important symptoms of SARS−COV-2 including headache, fatigue, shortness of breath fatigue, fever, dry cough, decreased blood oxygen levels and coagulation in blood circulation [[10], [11], [12]]. However, recent evidences have shown that laryngopharyngeal problems (diarrhea, sore throat), pneumonia, symptoms of functional weakness in the gastrointestinal tract and impaired kidney and liver function have been reported by infected patients [[13], [14], [15]].

One of the most important benefits of social-distancing is the reduction in the likelihood of COVID-19 acute respiratory disease transmission and protection of healthy individual in exposure to SARS-CoV-2 patients [16]. Staying home, hand hygiene and physical distancing are the most important interventions to slow disease spread [17].

Death from corona virus in cancer patients is challenging given the competing risks in delivering care this patient [18,19]. In the world countries (North America, Europe and Asia) have the highest causes of death due to cancer [20]. Cancer patients with corona virus remain unknown on active oncologic treatment [21].

One of the main causes of death in the world is death cause of cancer [22,23]. In recent decades, the prevalence of cancer has been increasing and is one of the threats to human health [24,25]. Based on result studied infected with COVID-19 causes more severe complications in cancer patients compared to other healthy people living in the community [26]. A lack of knowledge about how treatment the COVID-19 is the main problem in prevention and handle this disease in the large volume of people on world [26]. SARS−COV-2 has created many problems in providing health care to cancer patients [26].

The purpose of this study was to survey the common information on effect of COVID-19 pandemic on the risk factors attributed to cancer patients.

## 2 Material and methods

### 2.1 Search strategy and inclusion criteria

The present review study was performed based on reviewing and comparing the results of various researches published in databases: Science Direct, PubMed, Google Scholar, Elsevier and BMJ (Table 1). In this study, the most important limitations we had in searching for articles were years of publication 2000–2021 and English language.

**Table 1:** Search terms and query results.

Term	Google Scholar	Elsevier	Science Direct	PubMed	BMJ	Unique results
SARS-COV-2	180	205	130	140	63	295
Cancer patients	163	151	122	82	41	162
COVID-19	89	115	80	77	35	180
Risk factors	105	96	58	63	25	88
Health effects	93	107	81	52	18	95
Cancer patients and COVID-19	61	56	40	39	7	35
COVID-19 and respiratory systems	52	115	79	48	14	84
Cancer patients and respiratory systems	32	54	21	19	10	41
Total	1075	580	330	405	87	980

‘COVID-19′, ‘Cancer patients and COVID-19′, ‘SARS−COV-2′, ‘Cancer patients’, ‘Risk factors’, ‘Health effects’, ‘COVID-19 and respiratory systems’ and ‘Cancer patients and respiratory systems’. In total, 60 relevant original and review papers have been reviewed to establish the possible link between COVID-19 and Cancer patients. In next stage, we deleted duplicate and same terms articles. Also, we added 28 articles based on citations made in sources.

### 2.2 Ethical approval

Ethics License of the present study was acquired from the Ethics Committee of Ahvaz Jundishapur University of Medical Sciences (Code of ethics: IR.AJUMS.REC.1399.755). According to the national guidelines, studies such as this do not require individual consent.

## 3 Results of review of the epidemiological literature

### 3.1 Major a transmission hypothesis of SARS-CoV-2

**Fig. 1:**
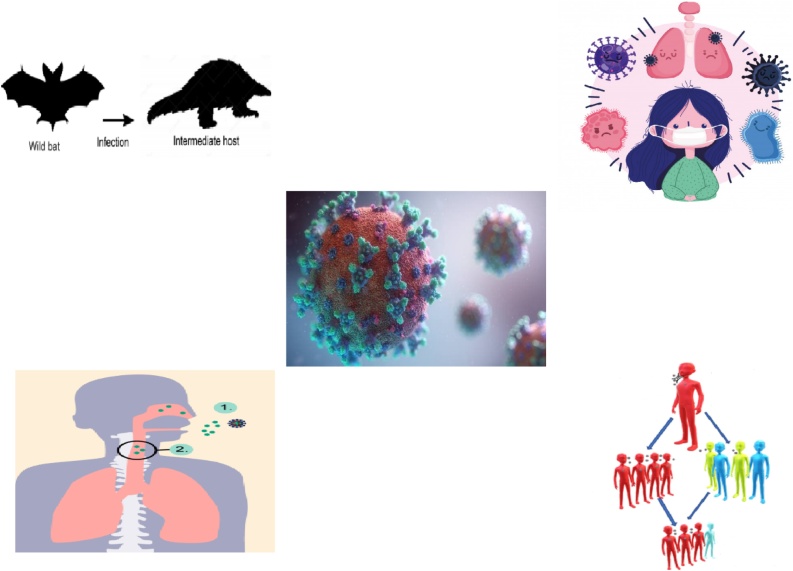
Schematic mechanism of the transmission hypothesis of SARS-CoV-2.

The aerosols from expired air coughs and sneezes people with symptoms and even asymptomatic SARS-CoV-2 people contaminate the immediate environment that can be the source of infection [[27], [28], [29], [30]]. One of the main agents that increasing the rate of transmission of SARS-CoV-2 is the high concentrations virus-containing aerosol in the air for long periods [31]. The main of animals which were transmission COVID-19 included snake, pangolins, avian, civet cats, camels, swine, phocine, bovine, reptiles, wild rabbits, crocodiles, canine, centipedes, goats, and frogs [32,33]. The potential intermediate host and a transmission hypothesis of COVID-19 pandemic is determined showed on Fig. 1.

Predominantly animal's causes coronaviruses and another types of it like SARS-CoV-2 transmission to humans [34]. Cell is the most important factor in spread infection disease such as COVID-19 [35].

### 3.2 Incidence of COVID-19

**Fig. 2:**
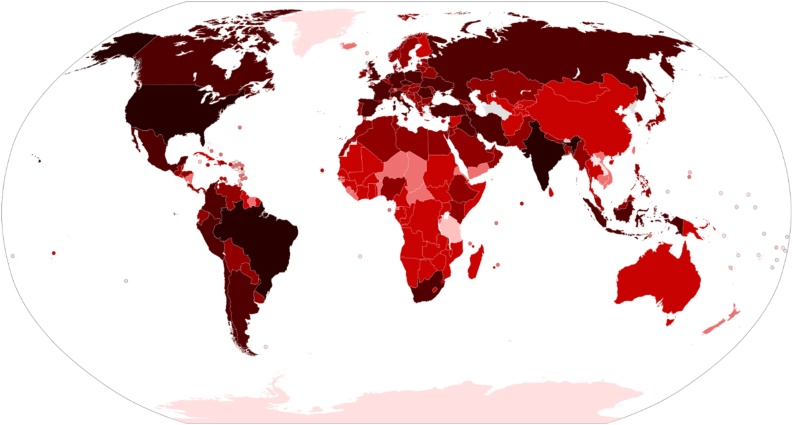
The incidence of COVID-19 in the world.

According to clinical observations and reports of the World Health Organization (WHO) and other health institutions around the world, the incidence and prevalence of SARS-CoV-2 is very different. But the point that all these centers have mentioned is that the causative agent of this disease shows the symptoms of the disease in the interval between 5–7 days and causes an increase in mortality. The geographical definition of the regions relation to the incidence of COVID-19 in the world showed in Fig. 2. Prevalence of SARS-CoV-2 on patients and information related to this disease is variable. Based on reported in December 2019 in China SARS-CoV-2 was diagnosed [36]. Covid-19 is an acute respiratory infectious disease [37]. The WHO has declared the infection a pandemic [37]. Based on reported the US Centers for Disease Control and Prevention (CDC) COVID-19 was recognized a serious public health threat [38,39]. Increasing rapidly the cases of disease and consequent increase in deaths rates causes by SARS-CoV-2 in worldwide due to the most affected by the disease [33,36]. COVID-19 cases with mild symptoms, recovered after 7–10 days of hospitalization [33]. According to result of several study incidence rates of COVID-19 reported 1 and 4 percent [17,18,40]. Higher risk among patients with lung or hematologic malignancies and COVID-19 in most studies showed [41]. Tests available for COVID-19 diagnosis, the molecular methods, is Real-time Polymerase Chain Reaction (PCR) [33]. The main characteristics of RT-PCR include viral genome detects, identifies mutations, follow the outbreak of the infection disease, have higher speed detect in compare to another molecular tests and the more advantageous economically [42]. Sometimes the Real-time Polymerase Chain Reaction test result may be negative, so it is necessary to check the patient's clinical evidence in medical centers [42].

Respiratory droplets, human-to-human, aerosol and direct contact are the most transmission routes in humans for the SARS-CoV-2 [39,43]. The main of animals which were transmission SARS-CoV-2 included snake, pangolins, avian, reptiles, swine, phocine, cicades, bovine, camels, canine, centipedes, goats, frogs, wild rabbits, crocodiles and kangaroos [32,33]. According to the result studies, the recovery and the global death rate are approximately 45 % and 6%, respectively [39].

### 3.3 Cancer patients and incidence of COVID-19

**Fig. 3:**
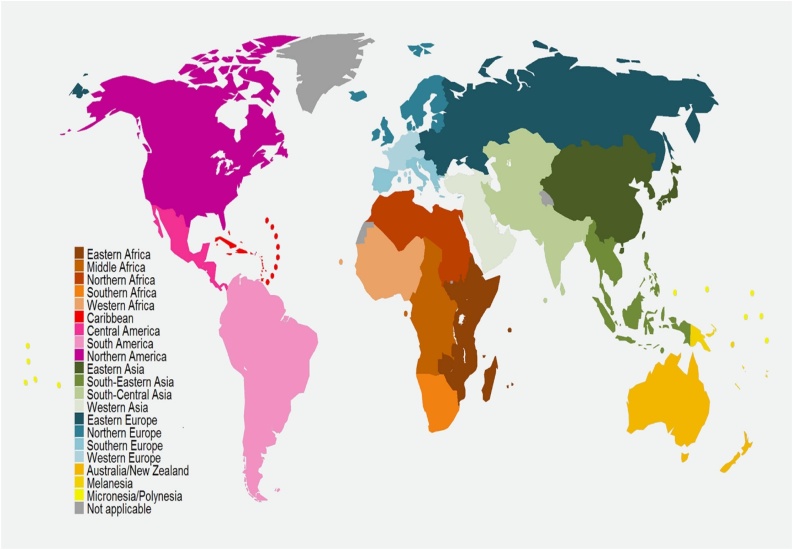
Global map incidence and mortality burden of cancer showing the 20 world regions [46].

Incidence of COVID-19 on cancer patients and information related to this disease is variable. According to result of several study incidence rates of COVID-19 among cancer patients reported 1 and 4 percent [17,18,40]. The population concurrently challenged by cancer and COVID-19, as the infection becomes more widespread, will undoubtedly expand asymmetrically across different geographies and risk cohorts [17,18]. Higher risk among patients with lung or hematologic malignancies and COVID-19 in most studies showed [41]. On patients with hematologic cancer the mortality ratio was 2.04 in the entire population, 3.72 among those younger than 70 years of age and 1.71 in people over age 70 [17,44,45]. The geographical definition of the regions relation to the incidence and mortality burden of cancer in each country showed in Fig. 3.

### 3.4 Clinical Cancer and COVID-19 patients

headache, sore throat, chills, muscle pain, dry cough, a loss of taste and smell, dyspnea, rigors, and fever are the most the clinical characteristics of COVID-19 in patients with cancer [47,48]. One of the clinical signs of coronavirus, which can threaten the immune system is anxiety, meanwhile, stress, anxiety and fear [39,49]. Implemented properly standard personal protective equipment (PPE) is the most imporyant ways for preventive of SARS-CoV-2 [50]. Based on result studies a severe illness and death from COVID-19 is higher among adult patients chronic [51,52].

According to result different studies a severe illness and death from COVID-19 is higher among adult patients with cancer [51,52]. A higher mortality rate on cancer patients with coronavirus has been observed compared with those without cancer [52]. Patients with advanced cancer and those with progressive disease have been found to have a worse prognosis than those with localized disease [26,53].

### 3.5 SARS−COV-2 and Cancer

According to various reports from world health sources, animals are most likely transition the primary source of COVID-19 outbreak [32]. One of the main agents that increasing the rate of transmission of SARS-CoV-2 is the high concentrations virus-containing aerosol in the air for long periods [31]. Different cancer type's diagnosis is very hard and can cause fear and anxiety among people. Now, despite the SARS−COV-2 pandemic, the situation has become more difficult and complicated [54,55].

Different studies reported that patients with cancer have a higher risk of severe events compared with patients without cancer [26,56,57]. Admitted to the intensive care unit (ICU) and death are the most important consequence COVID-19 among cancer patients [56,58].

**Fig. 4:**
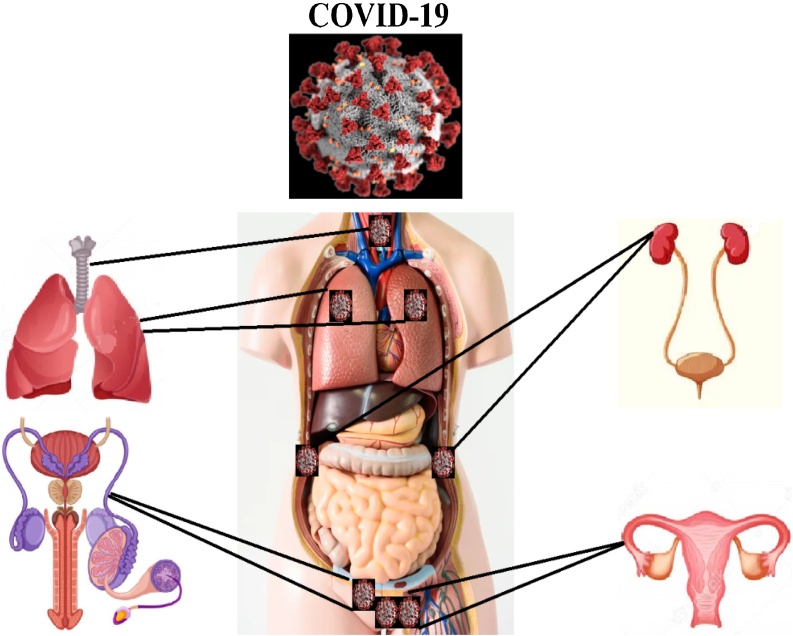
The body map of the risk of COVID-19 infection in cancer patents.

The body of cancer patents that are at high risk COVID-19 infection are (Lung adenocarcinoma (LUAD), Kidney Chromophobe (KICH), Prostate adenocarcinoma (PRAD), Esophageal carcinoma (ESCA), Uterine Corpus Endometrial Carcinoma (UCEC), and Kidney renal papillary cell carcinoma (KIRP) [59]. This body showed on Fig. 4. Due to the novelty of SARS−COV-2 and the lack of knowledge and studies on the treatment of cancer patients with SARS−COV-2, there are still no specific guidelines approved by the World Health Organization. Treatment of cancer patients with COVID-19 infection necessary for this patients.

The body map of the risk of COVID-19 infection in cancer patients showed in Fig. 4.

In addition Fig. 4 showed, the most important organs in cancer patents that are affected by SARS-CoV-2 infection are repository disease and lung failure, gastrointestinal manifestations, kidney diseases, renal dysfunction, urinary system and Ovaries and reproductive system [14].

### 3.6 Cancer patients and vaccination against SARS-CoV-2

The anticancer pharmacological, radiotherapeutic regimens, chemotherapeutic and long-term use immunosuppressive drugs and the resulting immunodeficiency are the main reasons increase risk catching SARS-CoV-2 in cancer patients [14].

Given that there are no common ways for infectious and viral diseases such as medication to prevent COVID-19, so A vaccine against SARS-CoV-2 especially in cancer patients is one of the most important ways to control and reduce the complications and eradicate this disease [34,60].

The immunosuppression of cancer patients is an extremely important risk factor including for inclusion / non-inclusion in clinical trials because of is very an important mention of vaccination against COVID-19 in cancer patients [34]. The effective vaccine is one of the main way eradicate the world from the SARS-CoV-2 [34,61].

Preventing COVID-19, decrease the severity of SARS-CoV-2, reduce the number of hospitalization cusses by coronavirus, optimal IgG anti-SARS-CoV-2 antibodies against COVID-19 after vaccination, significant reduction in hospitalization in the ICU, economical affordability and availability to all are among the most important features of a suitable and desirable vaccine [60].

Based on reported WHO Nucleic-acid vaccines (DNA and RNA vaccines), non-replicating viral vectors vaccines, virus vaccines (live attenuated and inactivated viruses) and protein-based vaccines are the main platforms for possible vaccine against COVID-19 [34,62,63].

## 4 Conclusion

The result our study present that SARS−COV-2 infection have a synergistic effect attributed to health of cancer patients. The results of various studies report that there is ample evidence of SARS−COV-2 infection with increased health endpoint among this patents. Oxidative stress in repository cells especially cancer cells is one of the most important side effects of COVID-19. According to what has been reported by pharmaceutical research centers around the world, an effective SARS−COV-2 drug has not yet been identified and produced.

Because of the lack of knowledge about to estimation of specified risk of corona virus for patients with cancer requires clear and uniform guidelines from the WHO and CDC.

The main susceptible to the infection are healthcare providers and the elderly with comorbidities. Therefore, preventive and inactive measures against the virus are necessary to stop and control the spread of the disease are very vital especially among patients with cancer. Therefore, Government Authorities at various level should put legislative framework for protect the residents against the risk of COVID-19 infection.

The investigation of SARS−COV-2 infection assessment among cancer patients is very vital for future studies. Reduction exposer to acute respiratory disease can decrease the health effects especially among cancer patients.

## Authorship contributions

Analysis or Interpretation: E.L., M.J.M.; Literature Search: M.J.M., H.Y.; Writing: E.L., M.J.M, and H.Y.

## Funding/Support

This work was financially supported by the Social Determinants of Health Research Center, Ahvaz Jundishapur University of Medical Sciences, Ahvaz, Iran (SDH-9939).

## Declaration of Competing Interest

No conflict of interest was declared by the authors.
